# Supplementary material for: Large-area integration of two-dimensional materials and their heterostructures by wafer bonding
Source: Nat Commun. 2021 Feb 10;12:917. doi: 10.1038/s41467-021-21136-0 (PMC7876008; doi:10.1038/s41467-021-21136-0)
Supplement: Supplementary file 1 — Supplementary Information [file 41467_2021_21136_MOESM1_ESM.pdf]

SUPPLEMENTARY INFORMATION

Large-Area Integration of Two-Dimensional Materials and Their Heterostructures by Wafer Bonding

Quellmalz et al.

Supplementary note 1: Integration of bottom electrodes to transferred graphene

Supplementary note 2: Fabrication of suspended graphene membranes

Supplementary note 3: Surface topography of Cu growth substrates

Supplementary note 4: Terahertz (THz) time-domain spectroscopy (TDS)

Supplementary note 5: Mobility and sheet resistance of integrated field-effect graphene devices

Supplementary note 6: Analysis of Raman spectroscopy of MoS_2 and BCB

Supplementary note 7: Analysis of photoluminescence spectroscopy of MoS_2

Supplementary note 8: Raman and photoluminescence spectroscopy of MoS_2/graphene heterostructures

Supplementary note 9: Formation and analysis of graphene/MoS_2 heterostructures

Supplementary References

Supplementary note 1: Integration of bottom electrodes to transferred graphene

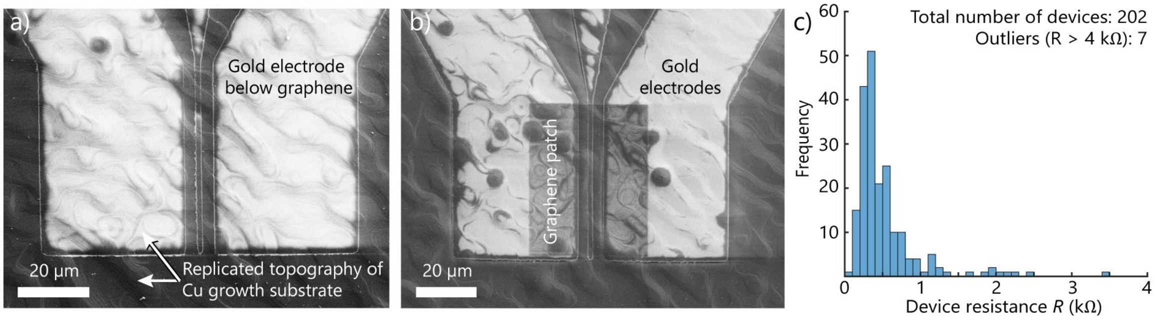

Supplementary Figure 1: Integrated bottom electrodes to transferred graphene. **a)** SEM image of graphene covering gold electrodes (bright) on bisbenzocyclobutene (BCB) after transfer and before structuring of the graphene layer. The topography of the copper (Cu) growth substrate is replicated in the BCB and metallization. **b)** SEM image of structured graphene patch (dark rectangle), connecting the three neighboring gold electrodes (bright) that rest on the BCB layer. **c)** Measured electrical resistances between the outer electrodes of 202 fabricated graphene devices featuring the device design shown in Supplementary Figure 2b).

To demonstrate the integration of transferred graphene with bottom electrodes, we spin-coated a 2.5 µm thick adhesive layer of bisbenzocyclobutene (BCB) (CYCLOTENE 3022-46, Dow Inc.) at 5000 rpm on the 100 mm diameter target wafer. Soft-bake on a hot-plate at 100 °C for 4 min removed solvents and solidified the adhesive layer. Pre-curing on a hot-plate in ambient atmosphere (190 °C, 30 min) partially cross-linked the BCB which increased its chemical stability. Consequently, the adhesive layer is resistant against solvents in the following process steps. The bottom electrodes were deposited and patterned on top of the BCB layer by a standard lift-off process and thermal evaporation (Spin-coating: ma-N 1420 negative tone photoresist (Micro Resist Technology GmbH) at 3000 rpm, soft-bake: 100 °C for 2 min, exposure: i-line stepper, development: ma-D 533 S (Micro Resist Technology GmbH), thermal evaporation: 20 nm Ti / 100 nm Au, lift-off: mr-Rem 700 XP (Micro Resist Technology GmbH)). Next, the target wafer was brought in proximity to a 4-inch sheet of monolayer CVD graphene on copper foil (Graphenea Inc.) with the graphene facing the BCB layer and the patterned electrodes. The substrate stack was then clamped into a bond fixture, without using spacers or flags and bonded in a commercial wafer bonder (Suss-SB8) at 190 °C for 20 min (ramp times: 20 min) using a bond force of 3 kN (bond pressure: 0.95 bar) in nitrogen atmosphere. Etching the copper foil in $FeCl_3$ solution and rinsing in deionized water uncovered the graphene, transferred to the target wafer and covering the electrodes (Supplementary Figure 1a). In a subsequent lithography step (Spin-coating: MICROPOSIT SPR700-1.2 positive tone photoresist (Dow Inc.) at 5000 rpm, soft-bake: 100 °C for 1 min, exposure: i-line stepper, PEB: 110 °C for 1 min, development: MICROPOSIT MF CD-26 (Dow Inc.)) and etching in O_2 plasma the graphene layer was patterned into graphene patches that connect the underlying bottom electrodes (Supplementary Figure 1b). To characterize the fabricated devices, we measured the electrical 2-terminal resistances between the outer electrodes of 202 devices at room temperature using a parameter analyzer (Keithley SCS4200, Tektronix, Inc.) (Supplementary Figure 1c).

Supplementary note 2: Fabrication of suspended graphene membranes

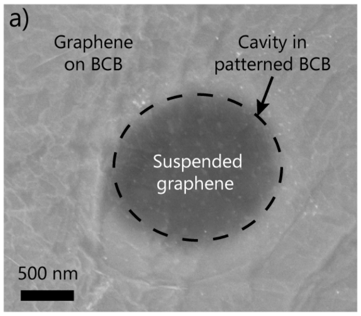

Supplementary Figure 2: SEM images of suspended graphene membrane over a circular cavity in patterned bisbenzocyclobutene (BCB).

To demonstrate the fabrication of suspended graphene membranes, we spin-coated a 2.5 µm thick adhesive layer of bisbenzocyclobutene (BCB) (CYCLOTENE 3022-46, Dow Inc.) at 5000 rpm on the 100 mm diameter target wafer. Soft-bake on a hot-plate at 100 °C for 4 min removed solvents and solidified the adhesive layer. Pre-curing on a hot-plate in ambient atmosphere (190 °C, 30 min) partially cross-linked the BCB which increased its chemical stability. Consequently, the adhesive layer is resistant against solvents in the following process steps. A standard lithography (Spin-coating: AZ 4533 positive tone photoresist (Microchemicals GmbH) at 4000 rpm, soft-bake: 100 °C for 1 min, exposure: contact lithography, development: MICROPOSIT MF CD-26 (Dow Inc.)) and dry etching of the BCB in O_2/SF_6 plasma (STS ICP Multiplex Advanced Oxide Etch system, O_2 flow: 15 sccm, SF_6 flow: 5 sccm, Coil power: 400 W, Platen power: 20 W, DC bias: 75 V, Process pressure: 1 mTorr), formed circular openings in the BCB layer. Next, the target wafer was brought in proximity to a 4-inch sheet of monolayer CVD graphene on copper foil (Graphenea Inc.) with the graphene facing the BCB layer with patterned cavities. The substrate stack was then clamped into a bond fixture, without using spacers or flags and bonded in a commercial wafer bonder (Suss-SB8) at 190 °C for 20 min (ramp times: 20 min) using a bond force of 3 kN (bond pressure: 0.95 bar) in nitrogen atmosphere. Etching the copper foil in $FeCl_3$ solution and rinsing in deionized water uncovered the graphene, transferred to the target wafer and suspended over the cavities that were etched in the BCB (Supplementary Figure 2).

Supplementary note 3: Surface topography of Cu growth substrates

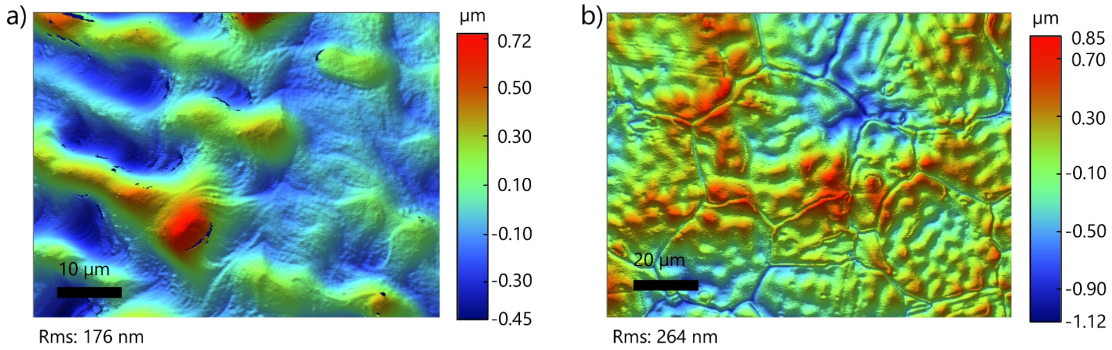

Supplementary Figure 3: White-light interferometric measurements of the microscopic surface topography of copper (Cu) substrates after material growth. **a)** Growth substrate of graphene (measurement area: 63 μm by 47 μm). **b)** Growth substrate of hexagonal boron nitride (measurement area: 125 μm by 94 μm). Root mean square (Rms).

Supplementary note 4: Terahertz (THz) time-domain spectroscopy (TDS)

While Raman measurements provide detailed knowledge about the local graphene quality, their point density is typically insufficient to characterize large-area graphene sheets as on full wafers. Contact based electrical characterization like the four-point probe method provide quantitative results of the sheet resistance but require potentially destructive mechanical contact to graphene and have a typical spatial resolution limit in the millimeter range. Eddy-current measurements are well suited for wafer-scale inspection, but the spatial resolution is even worse. As an alternative approach, THz Time Domain Spectroscopy has been demonstrated as a versatile tool for semiconductor inspection[1,2]. Typical measurement setups are equipped with THz emitters and detectors located in some cm distance to the sample, employing THz focusing optics that allow a diffraction limited spatial resolution between several 100 μm and a few mm. This already enables wafer-scale sheet resistance and mobility mapping, but can still be improved by switching to a THz near-field inspection scheme allowing to resolve features as small as a few 10 μm. Here, we used photoconductive (PC) near-field detectors based on free-standing cantilever microstructures made of low-temperature-grown Gallium Arsenide (LT-GaAs) which overcome the resolution limit of THz far-field setups[3]. The PC micro-probe detectors sample the amplitude and phase information of a THz pulse for temporally and spectrally resolved THz near-field imaging. From these THz-TDS information the electrical properties of a conductive thin-film can be obtained.

In this work, we did not operate the THz TDS near-field setup at its resolution limit of a few micrometers[3,4]. Instead, data acquisition was done with a step-size of a few 100 μm. Also, the distance of the near-field detector to the sample was set to a similar value. Thus, the probing area of each pixel was a few $100\ \mu\text{m}^2$, which is clearly larger than the intrinsic length scale that is probed by the THz spectroscopy. The intrinsic length scale is estimated by the distance a carrier moves during one cycle of the alternating THz field $l_\text{D}{\sim}\sqrt{D/\omega}$ where D is the diffusion coefficient

and $\omega = 2\pi f$ the angular frequency of the THz pulse[5]. The THz-spectrum of our setup covers the frequency range from $f = 0.1 - 3$ THz. In the following calculation we use the range $f = 0.5—1.5$ THz and the results from the vdP measurements to estimate the relevant length scales for the THz spectroscopy. Assuming similar mobility and charge carrier doping as extracted by the vdP measurements ($\mu = 2800\ \mathrm{cm^2V^{-1}s^{-1}}$ and $n = 3.7 \cdot 10^{12}\ \mathrm{cm^{-2}}$, respectively), we calculated the conductivity with the Drude formula

$$\sigma = en\mu = 1.66\ \mathrm{mS} \qquad (1),$$

and a mean free path of

$$l_{\mathrm{mfp}} = \sigma h/\left(2e^2\sqrt{\pi n}\right) \approx 63\ \mathrm{nm} \qquad (2)^6.$$

Accordingly, the scattering time of carriers is

$$\tau = l_{\mathrm{mfp}}/v_{\mathrm{F}} \approx 63\ \mathrm{fs} \qquad (3),$$

where $v_{\mathrm{F}} \approx 10^6\ m/s$ is the Fermi velocity[5]. Since the scattering time is smaller than the duration of the THz pulse ($\sim$1 ps), the movement of charge carriers is diffusive with a diffusion coefficient of

$$D = v_{\mathrm{F}} l_{\mathrm{mfp}}/2 \approx 314\ \mathrm{cm^2/s} \qquad (4)^7.$$

Hence, THz spectroscopy at 0.5—1.5 THz is sensitive to the average of the microscopic conductivity over the length scale

$$l_{\mathrm{D}} = \sqrt{D/\omega} \approx 60\text{—}100\ \mathrm{nm} \qquad (5).$$

Since both l_{D} and the l_{mfp} are smaller than the grain size, which is in the micrometer range, most carriers do not scatter on grain boundaries. Hence, our results from THz spectroscopy are a reasonable estimation of the average of the sheet conductivity in the area that is probed by the near-field detector.

We use THz time-domain spectroscopy (THz-TDS) to calculated the spatially resolved conductivity of graphene. Here, we illustrate the analysis based on two selected measurement points. One point on the reference sample area which is not covered by graphene (labeled as "Substrate" in Supplementary Figure 4d) and a second point on the graphene covered sample area (labeled as "Substrate + Graphene" in Supplementary Figure 4d). Note that thin layers of electrically insulating materials, such as nanometer-thick hBN, do not absorb THz radiation significantly and reference measurements with and without hBN layer did not show any difference. Hence, adding these layers to the substrate stack does not require a new reference measurement. Supplementary Figure 4d shows the two THz transmission curves together with the corresponding THz spectra as derived via Fast Fourier Transform (FFT) from the time domain data.

From the THz measurement data we computed the sheet conductivity of the graphene layer. This calculation followed the Tinkham formula (6), that relates the ratio of THz transmission through the substrate regions with graphene layer (T_{SL}) and the THz transmission through the reference substrate alone (T_{S}), to the sheet conductivity (σ_{sh}) of the graphene. The constant $Z_0 = 377\ \Omega$ is the freespace impedance, while n denotes the THz refractive index of the substrate with $n_{\mathrm{Si}} = 3.42$ [8].

$$T(\omega) = \frac{T_{\mathrm{SL}}(\omega)}{T_{\mathrm{S}}(\omega)} = \frac{1}{1 + \frac{\sigma_{\mathrm{sh}}(\omega) \cdot Z_0}{(n+1)}} \qquad (6)$$

During a measurement, we scanned the sample laterally in X- and Y-direction below the THz detector and recorded the amplitude of the THz transmission at each position. As a result of a complete THz mapping we obtained the spatially resolved sheet conductivity $\sigma(x, y)$ of the graphene, or the spatially resolved sheet resistance $R_{\text{sh}}(x, y) = 1/\sigma(x, y)$, as it is shown in the corresponding figures of the article.

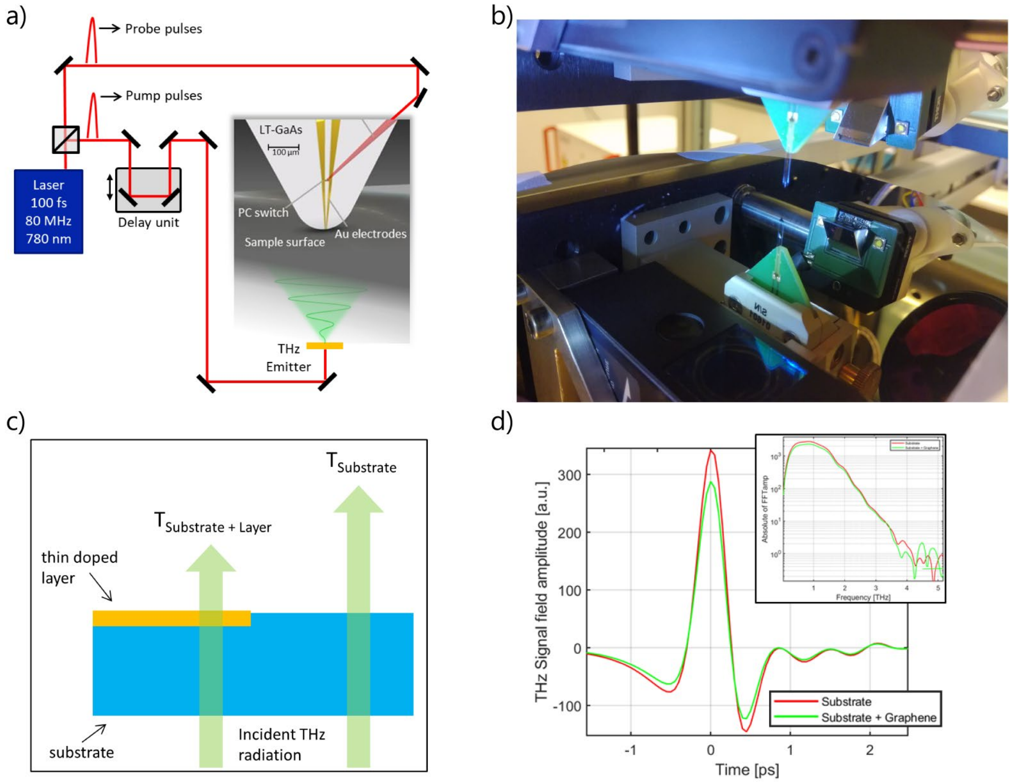

Supplementary Figure 4: Terahertz (THz) near-field spectroscopy of graphene. **a)** Schematic of the THz near-field transmission setup for characterization of the sheet resistance of graphene. **b)** Photograph of the measurement setup. **c)** Measurement scheme for conductive thin-films by THz near-field transmission spectroscopy. **d)** Exemplary data from THz time domain measurements and derived THz spectra (inset). The graphene sheet resistance is derived by comparing the signals from areas which are covered by graphene with signals from a reference area without graphene.

Supplementary note 5: Mobility and sheet resistance of integrated field-effect graphene devices

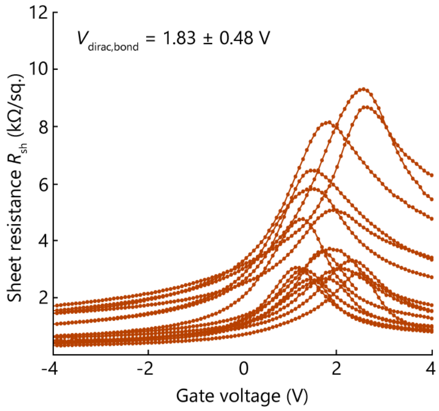

Supplementary Figure 5: Field-effect-graphene devices on BCB substrate fabricated by wafer bonding. Top-gate voltage dependency of the graphene sheet resistance of 16 graphene device at room temperature (same data set as for the histogram of the field-effect mobility in Figure 3d). The graphene was transferred by adhesive wafer bonding and is resting on the BCB layer in the final device (see Methods for details of the device fabrication and measurements). The sheet resistance reached a maximum at the bias voltage $V_{dirac,bond}$.

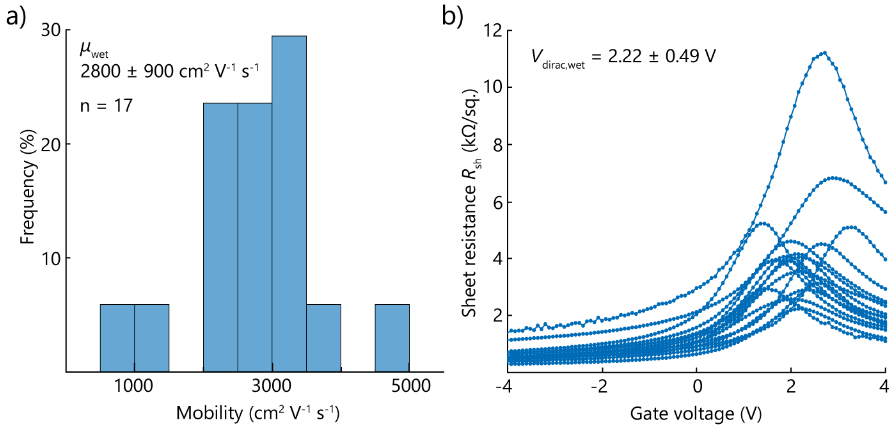

Supplementary Figure 6: Field-effect graphene devices on BCB substrate fabricated by conventional wet transfer. **a)** Histogram of the maximum filed-effect mobility (μ_{wet}) of 17 field-effect graphene devices at room temperature. **b)** Top-gate voltage dependency of the graphene sheet resistance (same data set as for the histogram in a)). The graphene was transferred by a wet transfer technique[9] and is resting on the BCB layer in the final device (see Methods for details of the device fabrication and measurements). The sheet resistance reaches a maximum at the bias voltage $V_{dirac,wet}$.

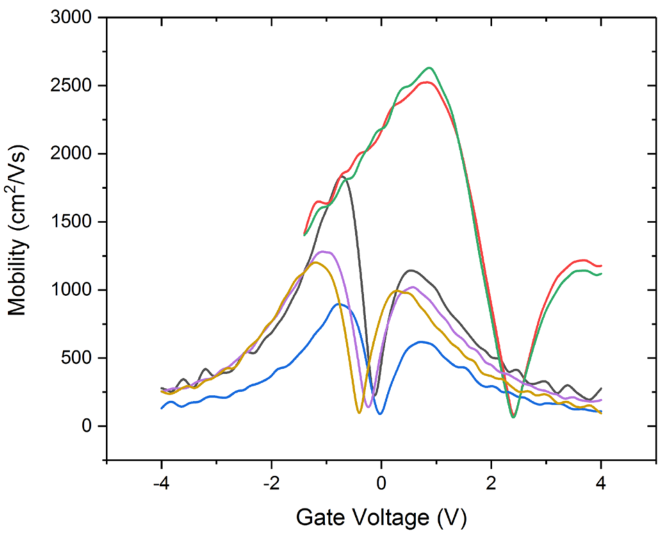

Supplementary Figure 7: Field-effect graphene devices on a quartz substrate fabricated by conventional wet transfer. Two-probe field-effect mobility of six top-gated devices on a quartz substrate at room temperature ($1700 \pm 700\ \mathrm{cm^2V^{-1}s^{-1}}$). The graphene was transferred by a conventional wet transfer technique[9] and is resting on the quartz substrate in the final device (see Methods for details of the device fabrication and measurements)

Supplementary note 6: Analysis of Raman spectroscopy of MoS_2 and BCB

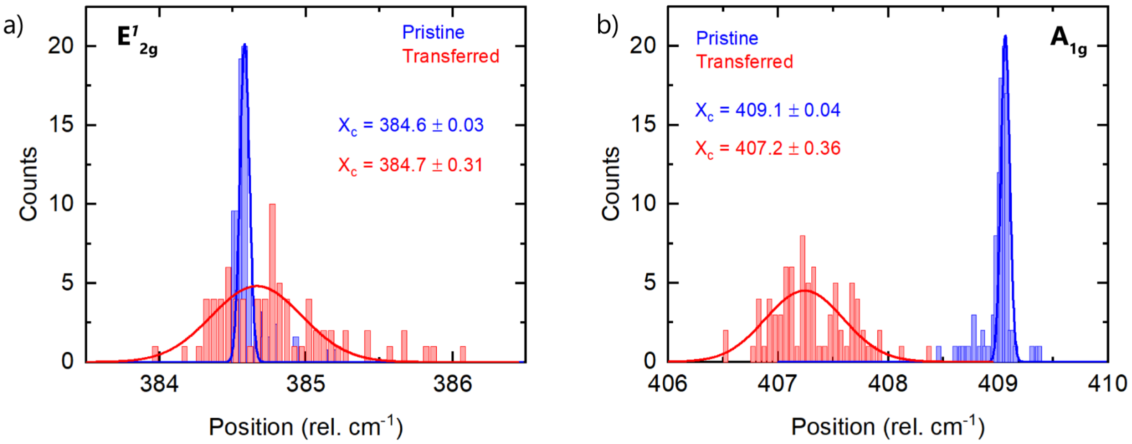

Supplementary Figure 8: Raman modes of molybdenum disulfide (MoS_2). **a)** Histograms of the E^{1}_{2g} mode position of pristine (blue) and transferred (red) MoS_2 films, extracted from area scans with 8 × 10 measurements in 1 mm^2 areas. Gaussian fits yield mode positions (X_c) of 384.6 cm^{-1} and 384.7 cm^{-1} for pristine and transferred MoS_2, respectively. The broadening of the E^{1}_{2g} position distribution is due to lower signal intensity which results in higher uncertainty of the fitting quality of the pseudo-Voigt function to the data. **b)** Histograms of the A_{1g} mode position of pristine (blue) and transferred (red) MoS_2 films, extracted from the same area scans as in a). Gaussian fits yield mode positions of 409.1 cm^{-1} and 407.2 cm^{-1} for pristine and transferred MoS_2, respectively. The A_{1g} mode is sensitive to changes in the substrate material which could cause the overserved shift of 1.9 cm^{-1} [10,11].

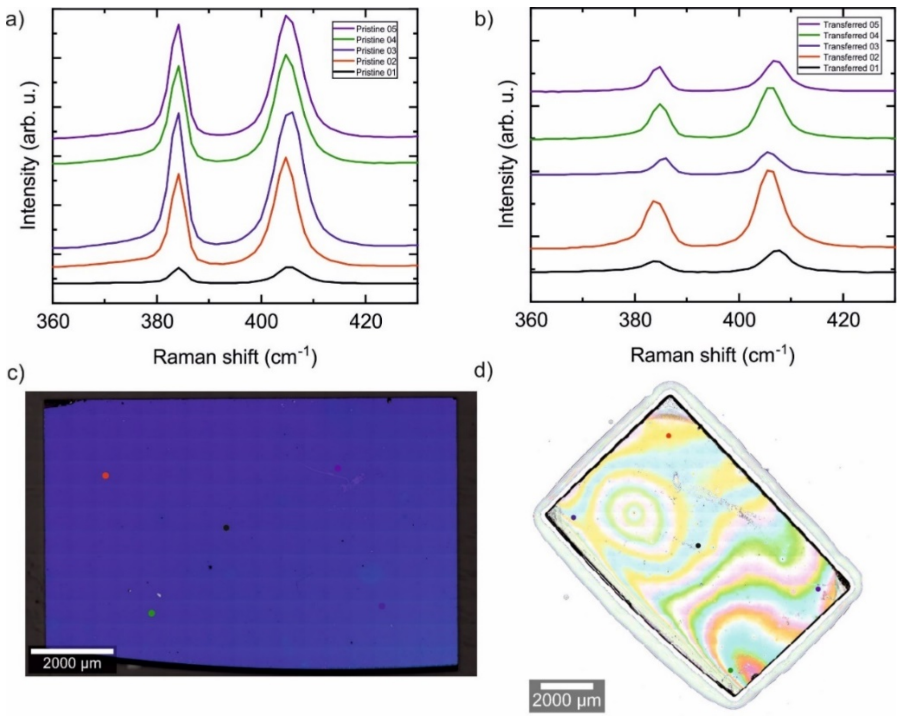

Supplementary Figure 9: Raman spectroscopy of molybdenum disulfide (MoS_2). **a)** Additional Raman spectra of MoS_2 on the SiO_2/Si growth substrate, measured at five different positions before transfer. **b)** Raman spectra of MoS_2 at five different positions after transfer to the silicon target wafer (placed on bisbenzocyclobutene (BCB)). **c)** Optical image of the as grown MoS_2 film on the oxidized silicon growth substrate. The colored dots indicate the positions of Raman measurements in a). **d)** Optical image of the transferred MoS_2 film on the silicon target wafer (placed on BCB). The colored dots indicate the positions of Raman measurements in b).

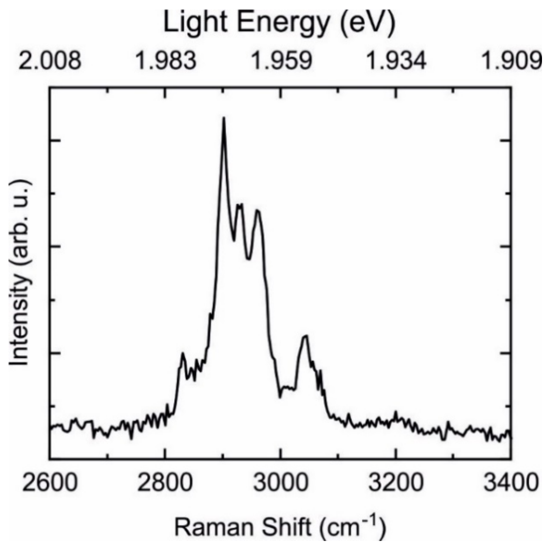

Supplementary Figure 10: Raman spectrum of a partially cross-linked bisbenzocyclobutene (BCB), measured on the same oxidized Si target wafer as used for the measurement in Figure 5a and 4b.

Supplementary note 7: Analysis of photoluminescence spectroscopy of MoS_2

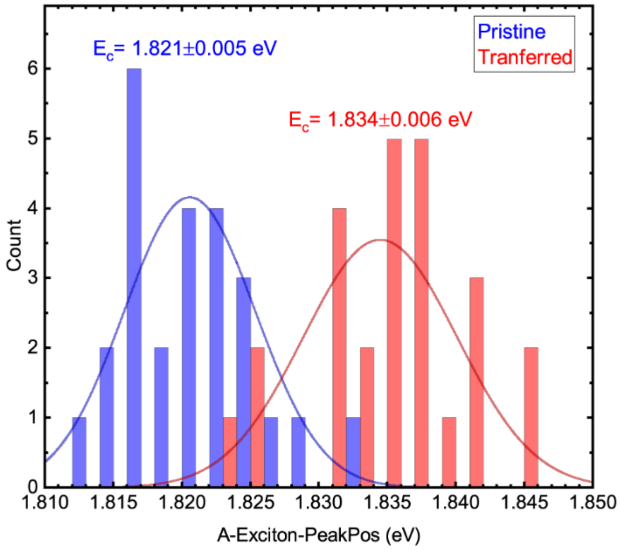

Supplementary Figure 11: Histogram of the peak positions of the A-excitonic transition in molybdenum disulfide (MoS_2) before (blue) and after transfer. After transfer, the mean position is blue shifted by 13 meV.

Supplementary note 8: Raman and photoluminescence spectroscopy of MoS_2/graphene heterostructures

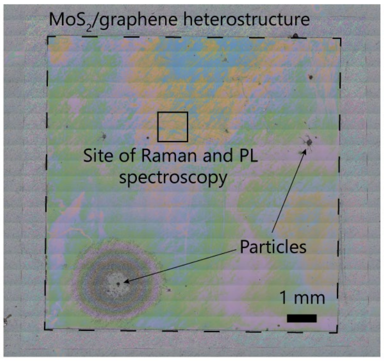

Supplementary Figure 12: Stitched white-light microscopy image with a molybdenum disulfide (MoS_2)/graphene heterostructure inside the dashed rectangle. The solid rectangle represents the acquisition site for Raman and photoluminescence (PL) spectroscopy. Particles on the growth substrate of the MoS_2 film caused partially bonded regions.

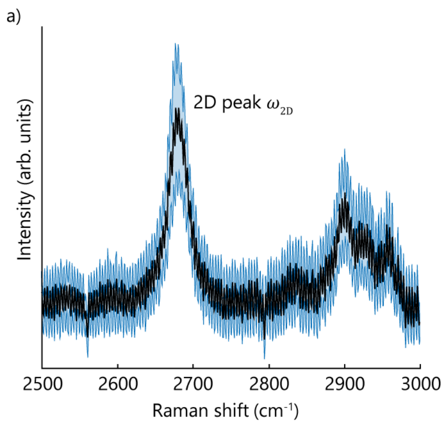

Supplementary Figure 13: Averaged Raman spectrum of graphene in the molybdenum disulfide (MoS_2)/graphene heterostructure (black) with standard deviation (blue). 2D peak position (ω_{2D}). The 225 individual spectra were acquired in an area of 1 mm^2 (same dataset as for the histograms in Figure 4d).

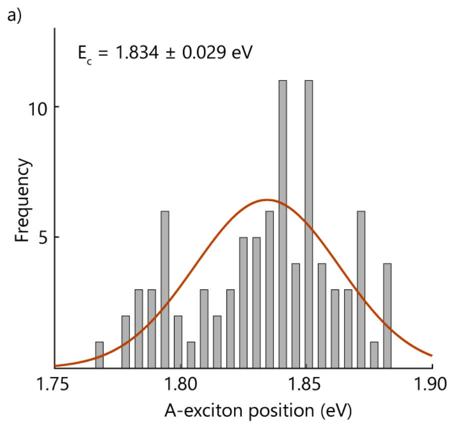

Supplementary Figure 14: Histogram of the peak positions (E_c) of the A-excitonic transition in molybdenum disulfide (MoS_2) in the MoS_2/graphene heterostructure after transfer. The 100 individual spectra were acquired in an area of 1 mm^2 (same dataset as for the histograms in Figure 4d) .

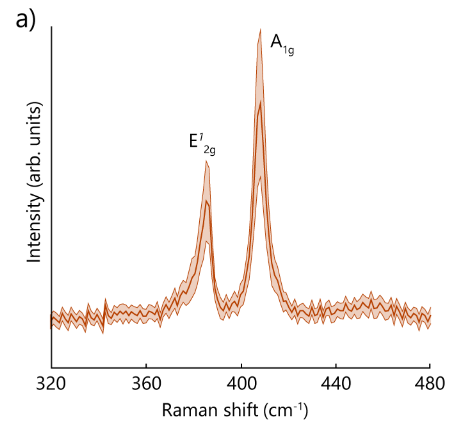

Supplementary Figure 15: Averaged Raman spectrum of molybdenum disulfide (MoS_2) in the MoS_2/graphene heterostructure with E^{1}_{2g} and A_{1g} modes. The 100 individual spectra were acquired in an area of 1 mm^2 (same dataset as for the histograms in Figure 4d) (shaded area: standard deviation).

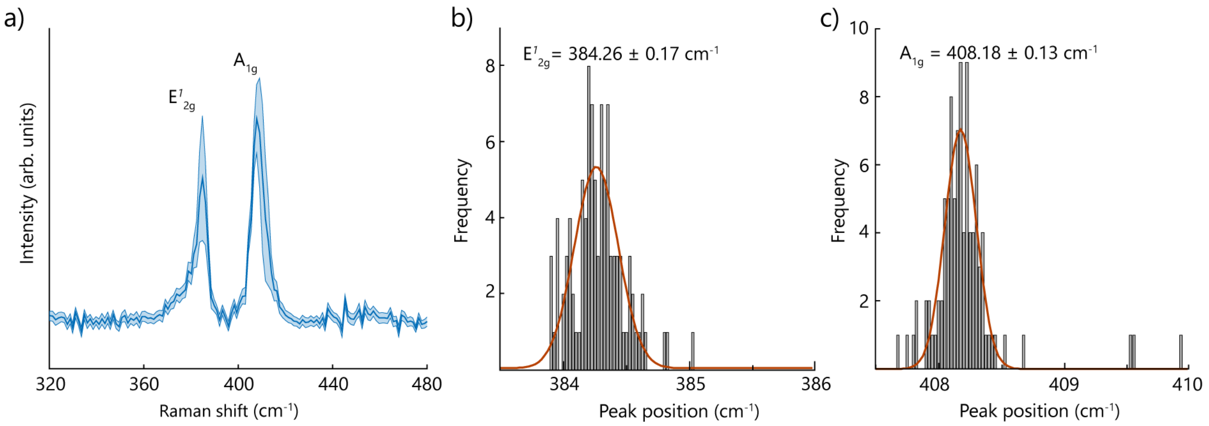

Supplementary Figure 16: Raman spectroscopy of molybdenum disulfide (MoS_2) on the growth substrate (SiO_2/Si chip) before forming the MoS_2/graphene heterostructure. **a)** Averaged spectrum (shaded area: standard deviation). **b)** Histograms of the E^{1}_{2g} mode position. **c)** Histograms of the A_{1g} mode position.

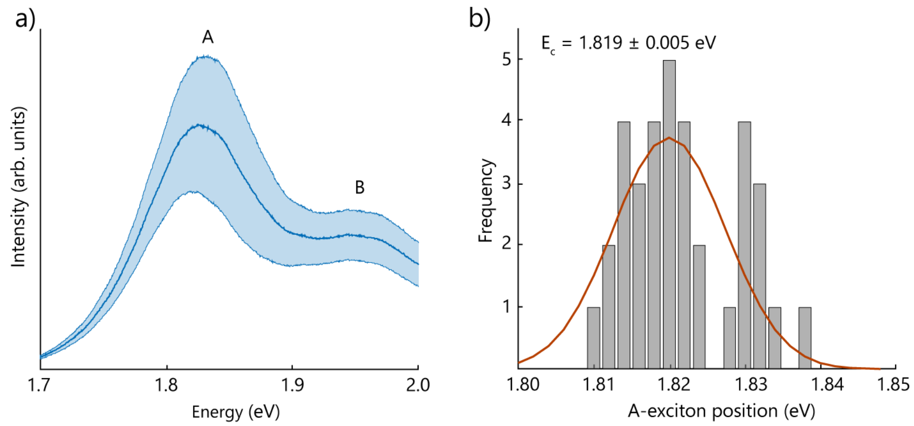

Supplementary Figure 17: Photoluminescence spectroscopy of molybdenum disulfide (MoS_2) on the growth substrate (SiO_2/Si chip) before forming the MoS_2/graphene heterostructure. **a)** Averaged PL spectrum (shaded area: standard deviation). **b)** Histogram of the peak positions (E_c) of the A-excitonic transition in MoS_2.

Supplementary note 9: Formation and analysis of graphene/MoS_2 heterostructures

First, the silicon target wafer (diameter: 100 mm, resistivity: > 10^4 Ω·cm) was spin-coated with a 2.5 µm thick layer of BCB (CYCLOTENE 3022-46 Dow Inc., spinning speed: 5000 rpm, soft-bake: 100 °C for 4 min). A MoS_2 film on a SiO_2/Si chip (10 mm by 10 mm, 2D Semiconductors Inc.) was placed on top of the adhesive layer (MoS_2 facing the BCB) and surrounded by silicon dummy chips to ensure a wider distribution of the forces over the wafer during the following bonding process. Bonding at 190 °C for 30 min attached the MoS_2/SiO_2/Si chip to the target wafer (bond force: 750 N with a resulting bond pressure of 3 bar, nitrogen atmosphere). Etching in O_2/SF_6 plasma cleaned the edges of the chip while a resist mask protected the remaining surface of the target wafer (Spin-coating: MICROPOSIT SPR700-1.2 positive tone photoresist (Dow Inc.) at 4000 rpm, soft-bake: 100 °C for 1 min, exposure: proximity lithography, development: MICROPOSIT MF CD-26 (Dow Inc.)). Submersion of the bonded stack in acetone stripped the resist mask and subsequent submersion in KOH solution (2M) detached the growth substrate (SiO_2/Si chip) from the MoS_2 layer by permeation of KOH into the MoS_2/substrate interface. The MoS_2 layer remained transferred on top of the BCB on the target wafer. Next, a sheet of monolayer CVD graphene on copper foil (2 cm by 1 cm, Graphenea Inc.) was bonded to the target wafer with the graphene facing the MoS_2 layer (bond temperature: 190 °C, bond time: 30 min, bond force: 750 N resulting in a bond pressure of 2.9 bar, vacuum atmosphere). Etching of the copper foil and rinsing in deionized water for 30 min uncovered the transferred graphene, forming a graphene/MoS_2 heterostructure on top of the BCB on the target wafer.

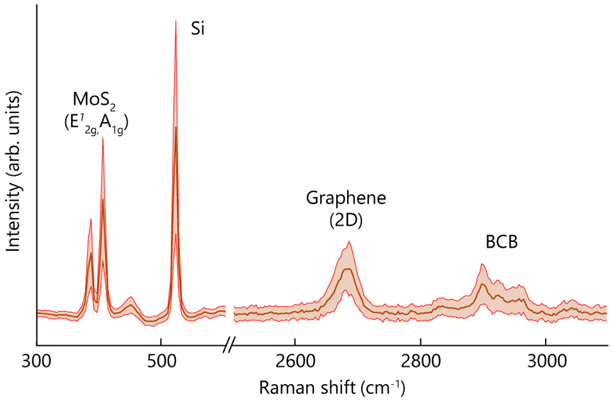

Supplementary Figure 18: Averaged Raman spectrum of the graphene/molybdenum disulfide (MoS_2) heterostructure, showing both the characteristic peaks of MoS_2 (E^{1}_{2g} and A_{1g}), graphene (2D), silicon (Si) and bisbenzocyclobutene (BCB) (shaded area: standard deviation).

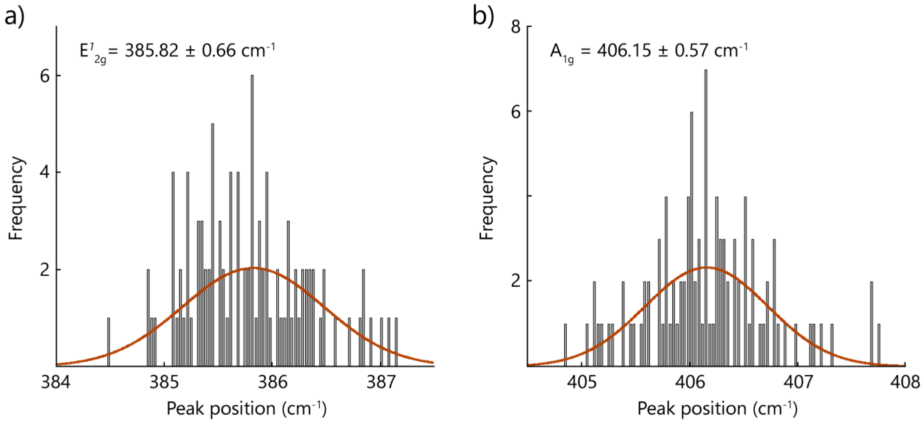

Supplementary Figure 19: Raman mode positions of molybdenum disulfide (MoS_2) in the graphene/MoS_2 heterostructures. **a)** Histogram of the E^{1}_{2g} mode position. **b)** Histogram of the A_{1g} mode position.

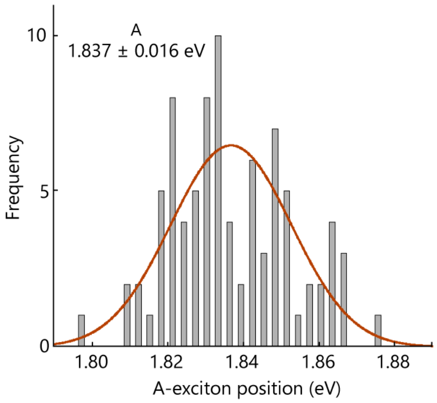

Supplementary Figure 20: Histogram of the peak positions of the A-excitonic transition in molybdenum disulfide (MoS_2) in the graphene/MoS_2 heterostructure.

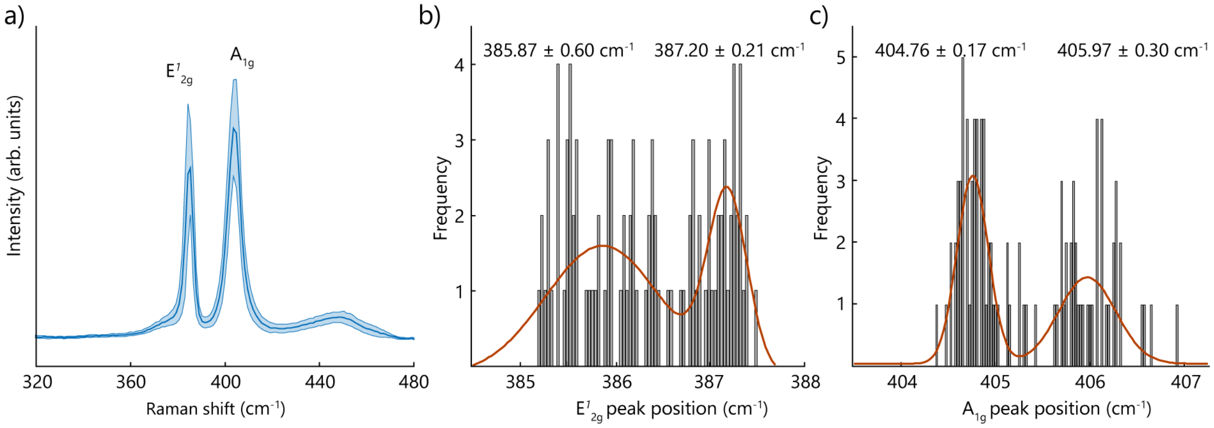

Supplementary Figure 21: Raman spectroscopy of molybdenum disulfide (MoS_2) on the growth substrate (SiO_2/Si chip) before forming the graphene/MoS_2 heterostructure. **a)** Averaged Raman spectrum (shaded area: standard deviation). **b)** Histogram of the E^{1}_{2g} mode position. **c)** Histogram of and A_{1g} mode position. The measurement captured two different regions of MoS_2, which resulted from a partly patchy MoS_2 growth. A double gaussian fit accounts for the different peak positions in these regions.

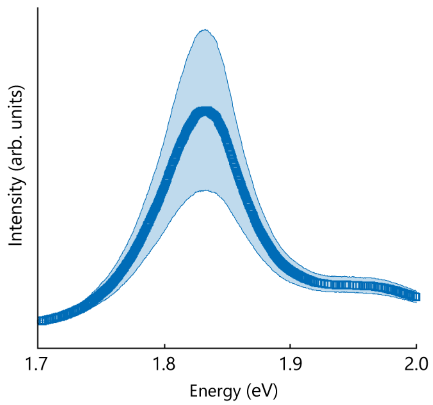

Supplementary Figure 22: Photoluminescence spectroscopy of molybdenum disulfide (MoS_2) on the growth substrate (SiO_2/Si chip) before forming the graphene/MoS_2 heterostructure.

Supplementary References

1. Tonouchi, M. Cutting-edge terahertz technology. *Nature Photonics* **1**, 97–105 (2007).
2. Herrmann, M., Tani, M., Sakai, K. & Fukasawa, R. Terahertz imaging of silicon wafers. *Journal of Applied Physics* **91**, 1247–1250 (2002).
3. Nagel, M., Matheisen, C. & Kurz, H. 12 - Novel techniques in terahertz near-field imaging and sensing. in *Handbook of Terahertz Technology for Imaging, Sensing and Communications* (ed. Saeedkia, D.) 374–402 (Woodhead Publishing, 2013). doi:10.1533/9780857096494.2.374.
4. Wächter, M., Nagel, M. & Kurz, H. Tapered photoconductive terahertz field probe tip with subwavelength spatial resolution. *Appl. Phys. Lett.* **95**, 041112 (2009).
5. Bøggild, P. *et al.* Mapping the electrical properties of large-area graphene. *2D Mater.* **4**, 042003 (2017).
6. Wang, L. *et al.* One-Dimensional Electrical Contact to a Two-Dimensional Material. *Science* **342**, 614–617 (2013).
7. Rengel, R. & Martín, M. J. Diffusion coefficient, correlation function, and power spectral density of velocity fluctuations in monolayer graphene. *Journal of Applied Physics* **114**, 143702 (2013).
8. Tinkham, M. Energy Gap Interpretation of Experiments on Infrared Transmission through Superconducting Films. *Phys. Rev.* **104**, 845–846 (1956).
9. Uzlu, B. *et al.* Gate-tunable graphene-based Hall sensors on flexible substrates with increased sensitivity. *Scientific Reports* **9**, 18059 (2019).
10. Buscema, M., Steele, G. A., van der Zant, H. S. J. & Castellanos-Gomez, A. The effect of the substrate on the Raman and photoluminescence emission of single-layer MoS_2. *Nano Res.* **7**, 561–571 (2014).
11. Rahaman, M. *et al.* Highly Localized Strain in a MoS_2/Au Heterostructure Revealed by Tip-Enhanced Raman Spectroscopy. *Nano Lett.* **17**, 6027–6033 (2017).
